# Age-Dependent Behavioral and Synaptic Dysfunction Impairment Are Improved with Long-Term Andrographolide Administration in Long-Lived Female Degus (*Octodon degus*)

**DOI:** 10.3390/ijms24021105

**Published:** 2023-01-06

**Authors:** Carolina A. Oliva, Daniela S. Rivera, Angie K. Torres, Carolina B. Lindsay, Cheril Tapia-Rojas, Francisco Bozinovic, Nibaldo C. Inestrosa

**Affiliations:** 1 Center of Aging and Regeneration UC (CARE-UC), Departamento de Biología Celular y Molecular, Facultad de Ciencias Biológicas, Pontificia Universidad Católica de Chile, Alameda 340, Santiago 8331150, Chile; 2 Facultad de Educación, Universidad de Las Américas, República 71, Santiago 8370040, Chile; 3 GEMA Center for Genomics, Ecology and Environment, Facultad de Ciencias, Ingeniería y Tecnología, Universidad Mayor, Camino La Pirámide 5750, Huechuraba, Santiago 8580745, Chile; 4 Center for Applied Ecology and Sustainability (CAPES), Departamento de Ecología, Facultad de Ciencias Biológicas, Pontificia Universidad Católica de Chile, Alameda 340, Santiago 8331150, Chile; 5 Laboratory of Neurobiology of Aging, Centro de Biología Celular y Biomedicina (CEBICEM), Facultad de Medicina y Ciencia, Universidad San Sebastián, Lota 2465, Santiago 7510157, Chile; 6 Centro de Excelencia en Biomedicina de Magallanes (CEBIMA), Universidad de Magallanes, Punta Arenas 6210005, Chile; 7 Laboratorio de Neurosistemas, Departamento de Neurociencias e Instituto de Neurociencia Biomédica (BNI), Facultad de Medicina, Universidad de Chile, Independencia 1027, Santiago 8380453, Chile; 8 Centro Científico y Tecnológico de Excelencia Ciencia & Vida, Avda. Zanartu 1482, Nunoa, Santiago 7780272, Chile

**Keywords:** *Octodon degus*, Andrographolide, aging, females, social memory, synaptic transmission, synaptic plasticity

## Abstract

In *Octodon degus*, the aging process is not equivalent between sexes and worsens for females. To determine the beginning of detrimental features in females and the ways in which to improve them, we compared adult females (36 months old) and aged females (72 months old) treated with Andrographolide (ANDRO), the primary ingredient in *Andrographis paniculata*. Our behavioral data demonstrated that age does not affect recognition memory and preference for novel experiences, but ANDRO increases these at both ages. Sociability was also not affected by age; however, social recognition and long-term memory were lower in the aged females than adults but were restored with ANDRO. The synaptic physiology data from brain slices showed that adults have more basal synaptic efficiency than aged degus; however, ANDRO reduced basal activity in adults, while it increased long-term potentiation (LTP). Instead, ANDRO increased the basal synaptic activity and LTP in aged females. Age-dependent changes were also observed in synaptic proteins, where aged females have higher synaptotagmin (SYT) and lower postsynaptic density protein-95 (PSD95) levels than adults. ANDRO increased the N-methyl D-aspartate receptor subtype 2B (NR2B) at both ages and the PSD95 and Homer1 only in the aged. Thus, females exposed to long-term ANDRO administration show improved complex behaviors related to age-detrimental effects, modulating mechanisms of synaptic transmission, and proteins.

## 1. Introduction

Andrographolide (ANDRO) is a diterpenoid lactone and the main active constituent of the *Andrographis paniculata*, a traditional Asian medicinal plant [1]. Its described effects include anti-inflammatory, antiviral, antitumoral, and antioxidant properties, most occurring at the systemic level [2]. However, the effect of ANDRO has also been evidenced in the central nervous system [3].

Among its multiple effects, ANDRO protects and benefits brain function, refining memory mechanisms and cognitive performance tasks in rodent models [3,4,5,6]. ANDRO prevents neuroinflammation-associated neurodegeneration in the prefrontal cortex and hippocampus of mice, improving working memory performance and regulating the expression of synaptic plasticity markers [7]. Due to these effects, several studies have been conducted with ANDRO in animal models of neurodegenerative diseases. In the mouse model of Alzheimer’s disease (AD), the double transgenic mouse model APP/PS-1, the treatment of 7-month-old but not 12-month-old mice with ANDRO for one month significantly decreased the Amyloid-β (Aβ) aggregates. These data suggest that ANDRO may prevent Aβ production and aggregation in the early stages of disease development [6]. In contrast, ANDRO reduced tau phosphorylation at both ages. At the same time, the synaptic proteins, synaptic plasticity paradigms (long-term plasticity, LTP), and spatial memory performance were improved with the treatment with ANDRO. These effects correlate with the increment of β-catenin and the inactive form of GSK-3β, indicating that ANDRO activates Wnt signaling [6]. In the same transgenic mice, ANDRO almost reverted their cognitive impairment, reduced oxidative stress, and protected mitochondria from instability [8]. Studies with the early-onset J20 Tg AD mouse model showed that four months of ANDRO treatment, applied at presymptomatic stages, improved some deficient metabolic markers, such as the lower levels of glucose uptake, ATP, and glycolytic rate, whereas by acting in the presynaptic region, it modulated the machinery to optimize the strength of the evoked response and improved cognitive performance [9]. These data suggest that developmental and disease progression times are relevant when using ANDRO as a treatment; timing is the essence of ANDRO treatment.

Exciting results have been obtained using ANDRO in the *Octodon degus*, an endemic Chilean rodent that develops metabolic, sleep, social-effective, and age disorders, including neurodegeneration, as in humans [5]. Degus is a diurnal, long-lived social animal that is an excellent model for studying high cognitive tasks [10,11]. Degus suffer from the effects of aging on several physiological aspects, including reduced cognitive function and deficient synaptic activity that start at around three years old and worsen over time. We recently reported sex differences in a study that involves age progression in degus [12]. Interestingly, we found that female degus are strongly affected by age. Young females (12–24 months old) have more efficient synaptic transmission than young males; however, aged females (60–84 months old) become several times less efficient than aged males. Studying LTP, we observed that males and females displayed the same amount of LTP, but aged females had to recruit more axonal terminals to produce the same evoked response. Instead, males recruited the same axonal terminals when young or aged, indicating that their synaptic mechanisms were not affected during age progression. These data showed a strong aging impact on the synaptic mechanisms of female degus [12].

Previous studies showed that aged female degus (56 months old) treated with ANDRO for three months increased memory recognition, learning performance, and the level of some postsynaptic proteins, reducing the aggregation of oligomeric Aβ species [5]. Although ANDRO also improved their basal synaptic activity, it was insufficient to reestablish LTP in these aged animals [5], suggesting that a more prolonged ANDRO treatment might be necessary for females in this species.

The evidence suggests that dose, treatment duration, and the animal’s age at the onset of treatment are fundamental in determining the potential effect of ANDRO in the brain. Therefore, knowing that ANDRO might benefit several physiological aspects during age progression in degus, we asked whether longer treatments could improve the deleterious effects of age in both adult (36 months old) and aged (72 months old) female degus. Females are traditionally misrepresented in scientific studies; assuming that the main physiological features are common to all sexes and evolve in similar ways, males are supra-represented when they are used as representative of both sexes. Nevertheless, the evolution of psychosomatic features shows animal lifetime differences depending on sex. Given our previous observations in which age had a more detrimental effect on females than males, we used adult and aged female degus to study ANDRO administration, suggesting that it would improve cognitive impairment and increase the synaptic strength associated with aging. To our knowledge, this is the first time a long-term ANDRO administration study in a natural long-lived animal model has been conducted.

## 2. Results

### 2.1. Aging-Dependent Cognitive Impairment Is Recovered by Long-Term ANDRO Administration

**Figure 1:**
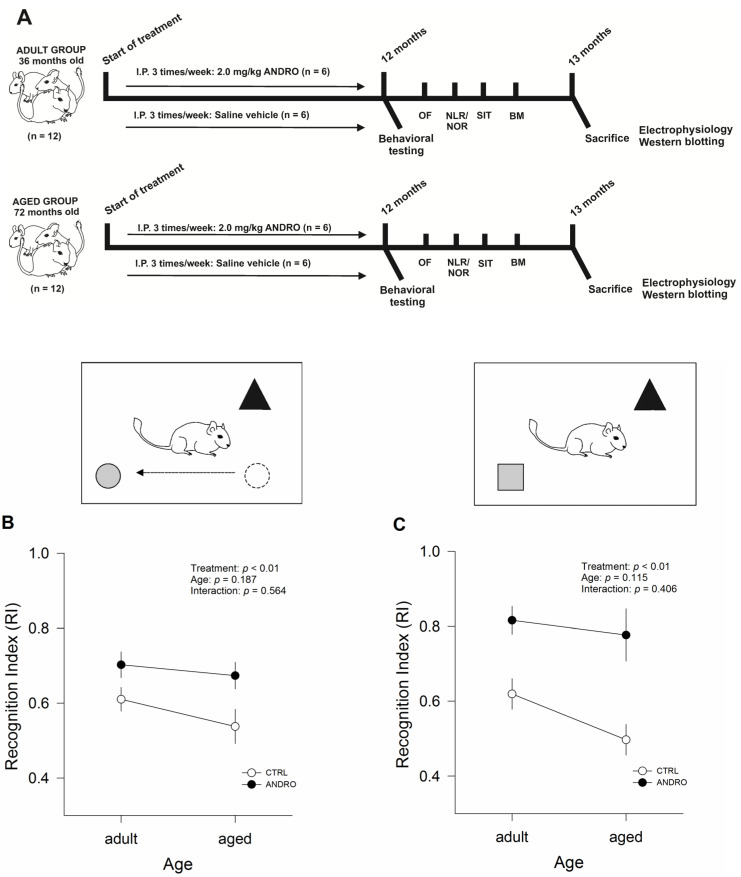
(**A**) Schematic diagram of the experimental design and procedures. Adult females (36 months old, n = 12) and aged female degus (72 months old, n = 12) received intraperitoneal (I.P.) injections of 2.0 mg/kg of ANDRO or vehicle solution three times per week over 12 months. Within months 12–13, the animals were subjected to four behavioral tasks: the open-field (OF) test, the novel object/novel location recognition test (NLR/NOR), the three-chambered social interaction test (SIT), and the Barnes maze (BM). By the end of the 13th month, each animal was euthanized daily for electrophysiology and Western blotting analysis. (B-C) Effect of the long-term ANDRO administration on cognitive performance measured by NLR/NOR test in adult and aged female degus. (**B**) RI for NLR; (**C**) RI for NOR. The RI was calculated as the time spent with object B divided by the sum of the time spent with object B and object A. The data were analyzed statistically using two-way ANOVA followed by Fisher’s LSD post hoc test. The statistical effect of ANDRO treatment, age, and the interaction between the two factors are indicated at the top. Each symbol corresponds to data from a single-age treatment group, represented as the mean ± SEM obtained from n = 6 adult females, n = 6 adult females + ANDRO, n = 6 aged females, and n = 6 aged females + ANDRO.

The schedule of the entire animal procedure is summarized in Figure 1A. Female degus were injected with ANDRO for 12 months, after which we executed behavioral testing. To evaluate the general state of animals, we performed the open-field test. The time spent in the corners and in the central zone of the arena, the number of central crossings, speed, and total distance traveled were not affected by the treatment or age, and no interaction was found between the two factors (all *p* > 0.05), suggesting that general behavior and locomotion are not affected or influenced by age, ANDRO administration, or the interaction between both factors (See Table S1 and Figure S1 in the Supplementary Materials).

We evaluated the impact of ANDRO on recognition memory in adult and aged degus, studying the exploratory motivation of degus to interact with a novel object in the novel location recognition/novel object recognition (NLR/NOR) test (Figure 1). Using the Recognition Index (RI), the two-way ANOVA analyses showed an effect of treatment (RI for NLR: F_(1,20)_ = 9.357, *p* < 0.01; RI for NOR: F_(1,20)_ = 23.63, *p* < 0.01), but not an effect of age (*p* = 0.187 and *p* = 0.115, respectively) or interaction between the two factors ((*p* = 0.0564 and *p* = 0.406; Figure 1B,C respectively). Further analysis showed that ANDRO-treated females had higher RI values than control animals, demonstrating that ANDRO improved recognition memory of features and location, as well as predilection for novel experiences in both ages. Though the adult and aged control showed no differences in this test (see Table 1), the results demonstrated that long-term ANDRO administration improved working memory. The time of interaction with a novel object and familiar object data are shown in the Supplementary Materials.

**Figure 2:**
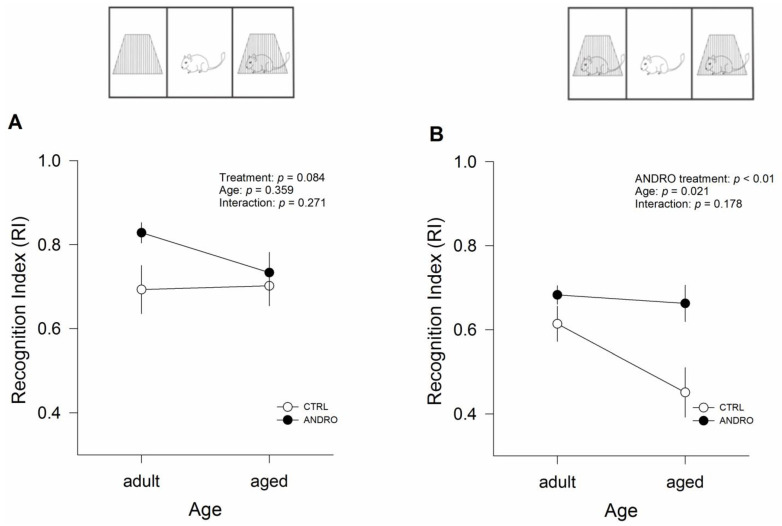
Effect of the long-term ANDRO administration on cognitive performance measured by the three-chamber social interaction test in adult and aged female degus. (**A**) RI for session 1 (social affiliation and sociability) expressed as the quotient of the time the subject degu spent with Partner I, divided by the sum of the time spent with Partner I and the empty wire cage. (**B**) RI for Session 2 (social memory and novelty) expressed as the quotient of the time the subject degu spent with Partner II divided by the sum of the time spent with Partners I and II. The data were analyzed statistically using two-way ANOVA followed by Fisher’s LSD post hoc test. The statistical effect of ANDRO treatment, age, and the interaction between the two factors are indicated at the top. Each symbol corresponds to data from a single-age treatment group, represented as the mean ± SEM obtained from n = 6 adult females, n = 6 adult females + ANDRO, n = 6 aged females, and n = 6 aged females + ANDRO.

Next, we performed three-chambered social interaction tests, a two-session test that evaluates sociability and social recognition memory. The RI for session 1, which assesses sociability, showed no effect of treatment (*p* = 0.084), age (*p* = 0.358), or interaction between the two factors (*p* = 0.271; Figure 2A). In contrast, the RI for session 2, which evaluates social recognition memory (Figure 2B), revealed an effect of treatment (F_(1,20)_ = 9.266, *p* < 0.01) and the age of animals (F_(1,20)_ = 6.239, *p* = 0.021). These differences were independent of the interaction between the two factors (*p* = 0.178; Figure 2B). Interestingly, the analysis showed that RI was significantly higher in the control adult females than in aged females (see Table 1 and Figure 2B), whereas ANDRO increased the RI in aged females, indicating a recovered condition due to the treatment. These data showed that neither age nor treatment affected the socialization of degus; however, ANDRO restored the social recognition memory that was impaired in aged female degus. The data relating to time interaction during session 1 and session 2 are shown in the Supplementary Materials.

**Figure 3:**
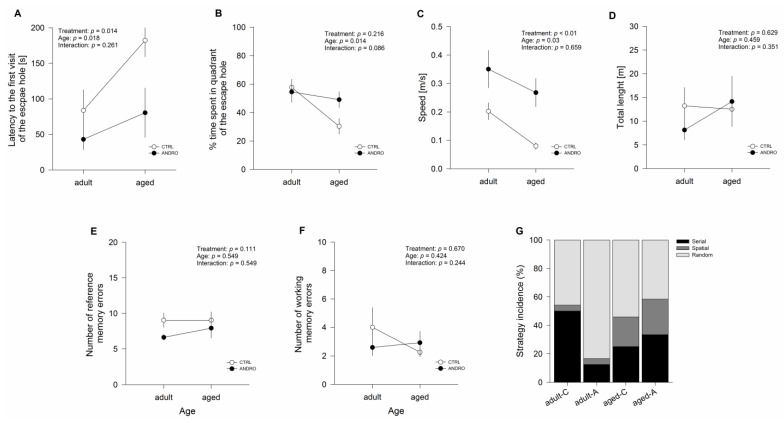
Effect of the long-term ANDRO administration on cognitive performance measured by the Barnes maze test in adult and aged female degus. (**A**) Latency to the first visit of the escape hole across test phase; (**B**) percentage of time spent in the quadrant (Q4) with the escape hole across test phase; (**C**) average speed across test phase; (**D**) total distance traveled on the maze across test phase € reference memory errors (every first visit of a non-escape hole in each trial) across test phase; (**E**) reference memory errors (every first visit of a non-escape hole in each trial) across test phase; (**F**) working memory errors (repeated visits to the same non-escape hole in the same trial) across test phase; (**G**) search strategies used for control and ANDRO adult and aged female degus during the test phase. The data were analyzed statistically using two-way ANOVA followed by Fisher’s LSD post hoc test. The statistical effect of ANDRO treatment, age, and the interaction between the two factors are indicated at the top. Each symbol corresponds to data from a single-age treatment group, represented as the mean ± SEM obtained from n = 6 adult females, n = 6 adult females + ANDRO, n = 6 aged females, and n = 6 aged females + ANDRO.

Aging is associated with a decline in selective aspects of cognitive performance together with brain functional and anatomical changes. A crucial cognitive function that declines with age is spatial navigation. To assess this function, the animals performed the Barnes maze test to examine the effect of treatment on spatial learning and memory processes. The data about the latency to find the escape hole, the number of references, memory errors, and the navigation strategy during the training phase are shown in SM. During the test phase (Figure 3A), the time taken to find the escape hole showed an effect of treatment (F_(1,20)_ = 7.244, *p* = 0.014) and age (F_(1,20)_ = 6.594, *p* = 0.018), but it was not altered by the interaction recorded between the two factors (*p* = 0.261; Figure 3A). Additional comparisons between groups showed that aged females took twice as long as adults to find the escape hole (Figure 3A and Table 1), but ANDRO reduced that time to half. We also measured the percentage of time spent in the quadrant. We found that it was not affected by treatment (*p* = 0.216) but had a significant effect due to age (F_(1,20)_ = 7.308, *p* = 0.013), and it was not altered by the interaction between both factors (*p* = 0.086; Figure 3B). Followed analysis showed that adult female degus spent more time than aged degus near the escape hole, suggesting that they can recall the location by staying in its proximity. We also measured the speed of traveling and found that it was affected by treatment (F_(1,20)_ = 14.440, *p* < 0.01), by age (F_(1,20)_ = 5.347, *p* = 0.031), and not by the interaction between both factors (*p* = 0.659; Figure 3C). The subsequent analysis showed that ANDRO-treated females moved faster than females treated with a vehicle, whereas adult animals were faster than aged ones (see Table 1). However, the total distance traveled was not altered by treatment (*p* = 629), age (*p* = 0.459), nor was the interaction between the two factors (*p* = 0.351; Figure 3D).

In the case of the reference and working memory errors to find the escape hole (Figure 3E,F), which counts the first visit and a repeated visit to the same non-escape hole per trial, respectively, our results showed no effect of treatment (*p* = 0.111 and *p* = 0.670, respectively), age (*p* = 0.549 and *p* = 0.424, respectively), or the interaction between both factors (*p* = 0.549 and *p* = 0.244, respectively). We also compared the strategy taken by degus to find the escape hole (Figure 3G). We observed that adult control female degus mainly used a serial and random-oriented strategy (50% and 46%), compared to the random and serial-search strategy (83% and 13%) observed in aged females (Figure 3G). In contrast, adult and aged female degus treated with ANDRO used a more random strategy (54% and 42%, respectively); however, they also increased the spatial-oriented strategy in the same proportion as the serial-oriented pattern (adult: 21% and 25%, aged: 25% and 33%; respectively; Figure 3G). Therefore, these results suggest that long-term ANDRO treatment improved spatial learning and memory in aged animals during the test phase.

### 2.2. Aged-Dependent Performance in Electrophysiological Parameters

The animals were later used to evaluate physiological aspects in electrophysiological experiments ex vivo. We first compared the parameters between the two ages of control groups to evaluate how similar or different they were. To determine the effect of ANDRO on synaptic activity, we studied synaptic transmission in the well-known synapse formed by Schaffer collaterals in the CA1 hippocampal region in the adult control and ANDRO-treated female degus. We stimulated the collateral axons and recorded the evoked activity at the level of the CA1.

**Figure 4:**
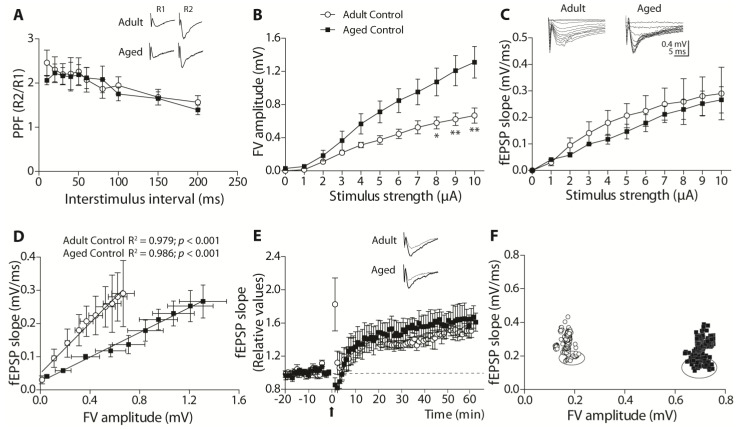
Comparison of synaptic transmission and plasticity mechanisms in adult and aged female degus. (**A**) The PPF plot shows the ratio of R2 versus R1 (R2/R1) slopes, recorded at several time intervals (20, 30, 50, 60, 80, 100, 150, and 200 ms). Inset: representative traces of R1 and R2 evoked fEPSPs separated by 20 ms. The I−O curves of fiber volley amplitudes (**B**) and fEPSP slopes (**C**) against stimulus intensity. Inset: representative traces of postsynaptic potentials evoked by increasing stimulus intensities (μA). Data analysis by two-way ANOVA followed by Bonferroni’s post hoc test. (**D**) The correlation plot of FV amplitudes and fEPSP slopes, each data adjusted by linear regression and analyzed by ANCOVA. (**E**) LTP plot shows the fEPSP slopes after TBS (arrow) normalized to the average of slopes before TBS. (**F**) Correlation plot of FV amplitudes and fEPSP slopes obtained before TBS (pre-TBS, symbols within circle) and after TBS (post−TBS, symbols alone). Comparison of 10 last min by *t*−test. Data represent the mean ± SEM obtained from n = 6 degus per group. At least three slices from each animal were considered replicates, averaged together, and counted as n = 1. * *p* < 0.05, ** *p* < 0.01.

We first analyzed a presynaptic form of plasticity, the paired-pulse facilitation (PPF), using a protocol that measures the release probability of synaptic vesicles from presynaptic terminals (Figure 4A). Two presynaptic spikes were evoked by stimulus separated at different times (20–200 ms). With the postsynaptic responses (fEPSP), called R1 and R2, we calculated a PPF index, as shown in Figure 4A. The adult and aged controls showed equivalent facilitation (ratio above 1) at the whole range of interval stimulus. The two-way ANOVA analysis showed a significant effect of the stimulus interval (F_(8,90)_ = 2.63, *p* < 0.01), but not age (*p* = 0.708) or the interaction between the factors (*p* = 0.98). This result shows that age alone does not affect the Ca^2+^-related vesicle release mechanisms (Figure 4A).

Then, we applied increasing current intensity levels to evoke increasing fEPSP slopes and FV amplitudes to build and analyze both pre- and postsynaptic components in an input–output (I-O) relationship. First, we plotted the FV amplitude against the stimulus amplitude (Figure 4B). The two-way ANOVA analysis showed a significant effect of age (F_(1,110)_ = 52.43, *p* < 0.001), stimulus (F_(10,110)_ = 23.93, *p* < 0.001), and no interaction between factors (*p* = 0.159), demonstrating that FV amplitudes are smaller in the tadult than in he aged control. Then, we plotted the evoked fEPSP slope against the stimulus amplitude and compared the control groups (Figure 4C). We found a significant effect of stimulus (F_(10,110)_ = 8.57, *p* < 0.001), but no effect of age (*p* = 0.101) and no interaction between factors (*p* = 0.996), indicating that in both groups, the evoked response was equivalent and did not depend on age.

To better determine the relationship between the recruited presynaptic terminal and the evoked postsynaptic response, we plotted the correlation between the FV amplitude and the fEPSP slope (Figure 4D). The data were adjusted to linear regression (adult slope = 0.378 ± 0.02, R^2^ = 0.979; aged slope = 0.186 ± 0.008, R^2^ = 0.986; Figure 4D). Thus, we performed an ANCOVA analysis to assess whether regression intercepts differed between groups. The analysis gave significant differences for intercepts (F_(1,17)_ = 56.65, *p* < 0.001), demonstrating that the regressions were different. This analysis demonstrated that adult female degus had more efficient basal synaptic transmission than aged females.

Next, we evaluated synaptic plasticity by studying long-term plasticity (LTP). We plotted the fEPSP slopes generated after TBS (arrow) relative to the basal activity before TBS in the adult and aged control groups (Figure 4E). As we could observe, adult and aged females generated a similar amount of LTP (comparison last 10 min: adults: 41.15 ± 11.12%; aged: 55.33 ± 16.43%, above the baseline; *t*-test_(10)_ = 0.715, *p* = 0.491). To determine whether the differences in the amount of elicited LTP were due to the synaptic status previous to TBS, we compared the FV amplitudes and fEPSP slopes of all the experiments used in the LTP analysis. We averaged them and plotted the correlations between these two parameters, as has been shown before [9,12,13]. In Figure 4F, we plotted the averaged FV amplitudes and slopes before (enclosed symbols) and after TBS (symbols alone) of adult control females (empty circles) and aged control females (solid squares). During basal stimulation, aged females recruited three times the number of axons (FV amplitude) than the adults (adults _FV_: 0.177 ± 0.026 mV, aged _FV_: 0.699 ± 0.016 mV; *t*-test_(10)_ = 17.1, *p* < 0.001) and evoked same responses (adults _fEPSP_: 0.217 ± 0.046 mV/ms, aged _fEPSP_: 0.187 ± 0.031 mV/ms; *p* = 0.601). During the post-TBS period, most of the recordings showed stable FV amplitudes before and after LTP induction, and the evoked responses were similar in both groups (*p* = 0.696; Figure 4F). These data show that despite the lower efficiency of aged synapses, there are mechanisms that can compensate for evoking the same postsynaptic activity and generating synaptic plasticity.

We observed that aged females were less efficient than adults but could compensate by evoking the same activity to cause similar LTP. Next, we compare both groups with the ANDRO-treated females to determine whether ANDRO could improve these features.

### 2.3. Long-Term ANDRO Administration in Electrophysiological Parameters

#### 2.3.1. ANDRO Reduces the Efficiency of Basal Synaptic Transmission in Adult Female Degus

**Figure 5:**
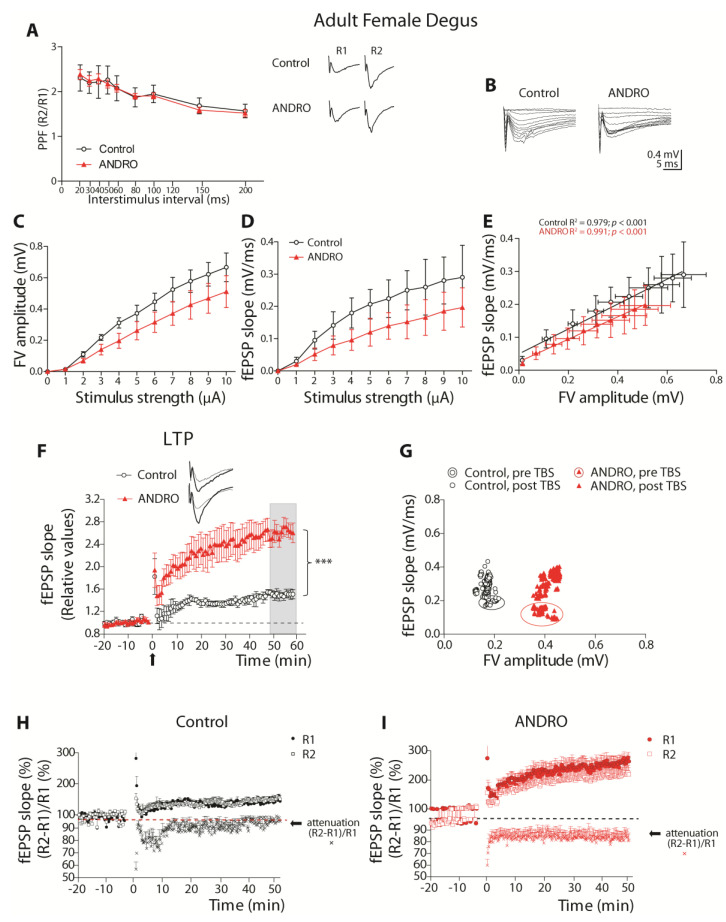
The effect of long−term ANDRO administration on synaptic transmission and plasticity in adult female degus. (**A**) The PPF plot shows the ratio of R2 versus R1 (R2/R1) fEPSP slopes, recorded at several time intervals (20, 30, 50, 60, 80, 100, 150, and 200 ms). Inset: representative traces of R1 and R2 evoked fEPSPs separated by 20 ms. (**B**) Representative traces of postsynaptic potentials evoked by increasing stimulus intensities (μA). The I−O curves of fiber volley amplitudes (**C**) and fEPSP slopes (**D**) against stimulus intensity. Data analysis by two-way ANOVA followed by Bonferroni’s post hoc test. (**E**) Correlation plot of FV amplitudes and fEPSP slopes. Each group was adjusted by linear regression and analyzed by ANCOVA. (**F**) LTP plot shows the fEPSP slopes after TBS (arrow) normalized to the average of slopes before TBS. (**G**) Correlation plot of FV amplitudes and fEPSP slopes obtained before TBS (pre-TBS, symbols within circle) and after TBS (post−TBS, symbols alone). (**H**,**I**) Attenuation plot showing the fEPSP slopes of R1(%), R2(%), and (R2-R1)/R1(%). Comparison of 10 last min by *t*−test. Data represent the mean ± SEM obtained from n = 6 degus per group. At least three slices from each animal were considered replicates, averaged together, and counted as n = 1, *** *p* < 0.001.

The study of PPF showed that both the control and ANDRO-treated groups had almost equivalent facilitation (ratio above 1) at the whole range of interval stimulus. The two-way ANOVA analysis showed a significant effect of the stimulus interval (F_(8,90)_ = 26.19, *p* < 0.001), but not the treatment (*p* = 0.862) or the interaction between the factors (*p* = 0.944). This result shows that ANDRO treatment does not affect the Ca^2+^-related vesicle release mechanisms in adult females (Figure 5A).

Then, we studied the I-O relationship, first analyzing the FV amplitudes per stimulus intensity (Figure 5B,C). The two-way ANOVA analysis showed a significant effect of the treatment (F_(1,110)_ = 15.21, *p* < 0.001), stimulus [F_(10,110)_ = 25.33, *p* < 0.001], and no interaction between factors (*p* = 0.870), demonstrating that FV amplitudes were smaller in ANDRO than the control. Then, we plotted the evoked fEPSP slope against the stimulus amplitude and compared the control and ANDRO-treated groups (Figure 5B,D). We found a significant effect of treatment (F_(1,110)_ = 10.22, *p* < 0.001), stimulus [F_(10,110)_ = 5.45, *p* < 0.001], and no interaction between factors (*p* = 0.991). The input–output data indicated that fewer axons were recruited, and fewer responses were evoked in ANDRO than in the control, suggesting that ANDRO lowered the basal excitability in adult females.

The correlation between the FV amplitude and the fEPSP slope was adjusted to a linear regression (control _slope_ = 0.378 ± 0.02, R^2^ = 0.979; ANDRO _slope_ = 0.341 ± 0.011, R^2^ = 0.991; Figure 5E). Thus, we performed an ANCOVA analysis to assess whether the intercepts and the slopes differed between groups. The analysis gave significant differences for intercepts (F_(1,17)_ = 52.93, *p* < 0.001) but not for slopes (*p* = 0.157), demonstrating that the curves were significantly different. Altogether, this analysis demonstrated that in adult female degus, ANDRO lowered the efficiency of basal synaptic transmission compared to the control.

#### 2.3.2. ANDRO Increases the Long-Term Synaptic Plasticity in Adult Female Degus

We evaluated synaptic plasticity by studying LTP (Figure 5F). As we can observe, the treatment with ANDRO increased the amount of LTP in the control several times (comparison last 10 min: Control: 41.15 ± 11.12%; ANDRO: 161.47 ± 14.97%, above the baseline; t-test_(10)_ = 6.452, *p* < 0.001). Then, we compared the FV amplitudes and fEPSP slopes of all the experiments used in the LTP analysis (Figure 5G). We plotted the averaged FV amplitudes and slopes before (enclosed symbols) and after TBS (symbols alone) of control females (black circles) and ANDRO-treated female degus (red triangles). During basal stimulation, ANDRO-treated adult females recruited twice the number of axons (FV amplitude) than the control adults (control _FV_: 0.177 ± 0.026 mV, ANDRO _FV_: 0.338 ± 0.058 mV; *t*-test_(10)_ = 2.53, *p* < 0.029), but they evoked the same responses (control _fEPSP_: 0.217 ± 0.046 mV/ms, ANDRO _fEPSP_: 0.150 ± 0.046 mV/ms; *p* = 0.33). During the post-TBS period, most of the recordings showed stable FV amplitudes before and after LTP induction, and the evoked responses were similar in both groups (control _fEPSP_: 0.313 ± 0.066 mV/ms, ANDRO _fEPSP_: 0.327 ± 0.095 mV/ms; *p* = 0.91; Figure 5G). These data show that the LTP protocol did not evoke higher responses but recruited more axons in adult females with ANDRO than controls.

We also studied the attenuation phenomena, which is the effect of LTP on the presynaptic component during LTP development (Figure 5H,I). Two stimuli, R1 and R2, were evoked to generate presynaptic spikes separated by 50 ms before and after TBS. Hippocampal CA3-CA1 synapses are low-release probability synapses, where the second pulse releases more neurotransmitters than the first pulse (Figure 5A, inset). We then performed the same analysis for the LTP experiments to determine the effect of presynaptic sites on the plasticity effect. In Figure 5H,I, we plotted both the R1 and R2 slopes and the percentage of attenuation between them using the formula: (R2-R1)/R1. The attenuation analysis indicated that at the beginning of the LTP induction, the ratio R2/R1 decreased by the increment of R1. This effect was similar between the control and ANDRO-treated group (control: 66.04 ± 3.28%, ANDRO: 64.80 ± 4.75%; *p* = 0.834). However, the initial magnitude of attenuation does not last; its decay depends on how much the TBS affects the pre- or postsynaptic mechanisms. In the case of the adult females, the attenuation decays faster in the control group, because the magnitude of R1 returns to similar values before TBS, making the facilitation of the presynaptic component more prevalent. Instead, with ANDRO, the attenuation remains stable until the end of the recording, because the magnitude of R1 remains high; this suggests that the postsynaptic component becomes relevant (comparison last 10 min: Control: 93.89 ± 1.91%, ANDRO: 74.75 ± 5.32%; *t*-test_(10)_ = 3.39, *p* < 0.01). These data indicate that adult females have a different/altered mechanism that prevents the stability of postsynaptic response, a mechanism that ANDRO could restore.

#### 2.3.3. ANDRO Increases the Efficiency of Basal Synaptic Transmission in Aged Female Degus

**Figure 6:**
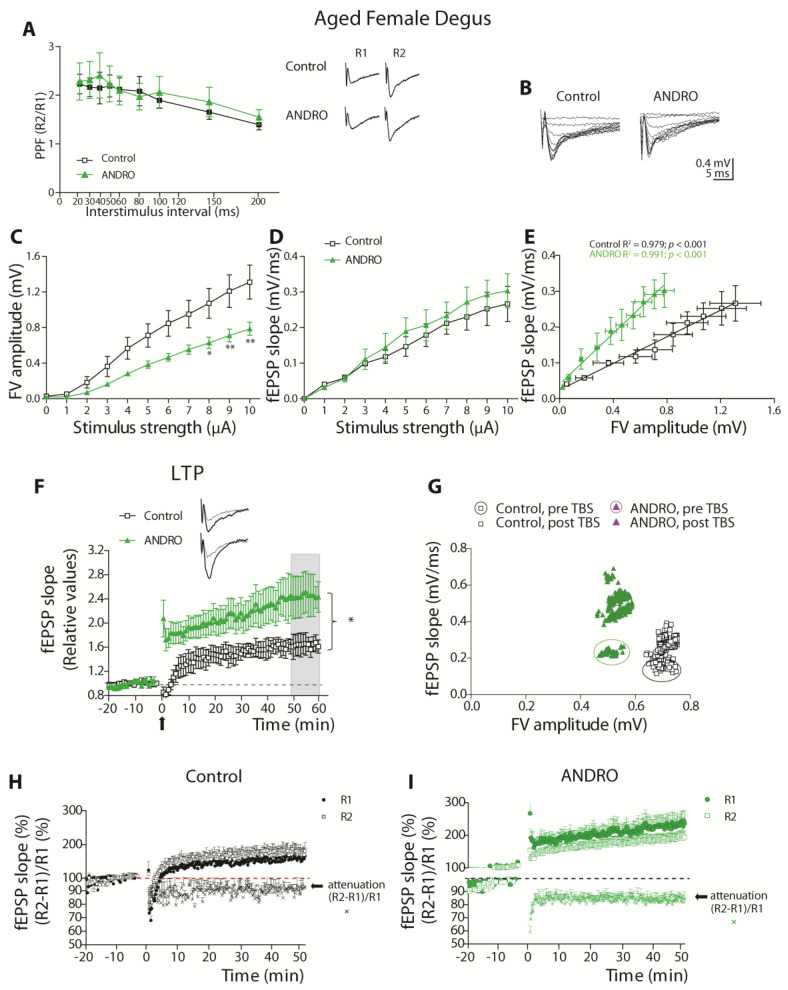
The effect of long-term ANDRO administration on synaptic transmission and plasticity in aged female degus. (**A**) The PPF plot shows the ratio of R2 versus R1 (R2/R1) fEPSP slopes, recorded at several time intervals (20, 30, 50, 60, 80, 100, 150, and 200 ms). Inset: representative traces of R1 and R2 evoked fEPSPs separated by 20 ms. (**B**) Representative traces of postsynaptic potentials evoked by increasing stimulus intensities (μA). The I−O curves of fiber volley amplitudes (**C**) and fEPSP slopes (**D**) against stimulus intensity. Data analysis by two-way ANOVA followed by Bonferroni’s post hoc test. (**E**) Correlation plot of FV amplitudes and fEPSP slopes. Each group was adjusted by linear regression and analyzed by ANCOVA. (**F**) LTP plot shows the fEPSP slopes after TBS (arrow) normalized to the average of slopes before TBS. (**G**) Correlation plot of FV amplitudes and fEPSP slopes obtained before TBS (pre-TBS, symbols within circle) and after TBS (post−TBS, symbols alone). (**H**,**I**) Attenuation plot showing the fEPSP slopes of R1(%), R2(%), and (R2−R1)/R1(%). Comparison of 10 last min by *t*−test. Data represent the mean ± SEM obtained from n = 6 degus per group. At least three slices from each animal were considered replicates, averaged together, and counted like n = 1, * *p* < 0.05, ** *p* < 0.01.

As shown in Figure 6A, the control and treated aged degus group showed a similar facilitation ratio (above 1) at the whole range of stimuli. The two-way ANOVA showed no significant effect of stimulus interval (*p* = 0.081), treatment (*p* = 0.395), or interaction between factors (*p* = 0.99; Figure 6A), indicating that no Ca^2+^-related vesicle release mechanisms are affected by ANDRO.

Then, we plotted the I-O relationship with fEPSP slopes and FV amplitudes. The two-way ANOVA analysis of the FV amplitude against stimuli amplitude (Figure 6B,C), showed a significant effect of treatment (F_(1,110)_ = 48.56, *p* < 0.001), stimulus [F_(10,110)_ = 28.08, *p* < 0.001], and no interaction between factors (*p* = 0.098). These data show that in the control, FV amplitudes were significantly higher, indicating that are more axons were recruited than in the ANDRO-treated group. Then, we plotted the evoked fEPSP slopes against the stimuli amplitude. The analysis showed a significant effect of stimulus (F_(10,110)_ = 19.16, *p* < 0.001), but not of treatment (*p* = 0.100) or the interaction between these factors (*p* = 0.996; Figure 6B,D), demonstrating that the evoked response was similar in the control and ANDRO-treated group. Like that observed in adult females, these data show significantly fewer axons recruited during stimulation in aged females with ANDRO compared to control.

We plotted the correlation between the FV amplitude and the fEPSP slope to determine the efficiency of the evoked response by activated axon (Figure 6E). The regression analysis showed a significant positive association between the fEPSP slopes and the fiber volley in both groups (control _slope_ = 0.186 ± 0.008, R^2^ = 0.986; ANDRO _slope_ = 0.356 ± 0.014, R^2^ = 0.988; Figure 6E). The covariance analysis showed significant differences for intercepts (ANCOVA _intercept_: control vs. ANDRO: F_(1,17)_ = 45.15, *p* < 0.001), demonstrating that the ANDRO group was more efficient; less axonal recruitment evoked the same response, improving basal synaptic transmission in aged female degus.

#### 2.3.4. ANDRO Increases the Long-Term Synaptic Plasticity in Aged Female Degus

We plotted the fEPSP slopes generated after TBS, relative to the basal activity before TBS (Figure 6F), in the control and ANDRO-treated groups. The results indicated that the treatment with ANDRO increased the amount of LTP compared to the control (comparison last 10 min: control: 55.33 ± 16.43%; ANDRO: 145.35 ± 34.63%, above the baseline; *t*-test_(10)_ = 2.349, *p* = 0.041). Then, to compare the FV amplitudes and fEPSP slopes, we plotted the averaged FV amplitudes and slopes before (enclosed symbols) and after TBS (symbols alone) of control females (black squares) and ANDRO-treated female degus (purple triangles) (Figure 6G). During basal stimulation, ANDRO-treated aged females recruited a lesser number of axons (FV amplitude) than the aged control (control _FV_: 0.699 ± 0.016 mV, ANDRO _FV_: 0.489 ± 0.073 mV; *t*-test_(10)_ = 2.81, *p* < 0.01) but evoked the same responses (control _fEPSP_: 0.187 ± 0.031 mV/ms, ANDRO _fEPSP_: 0.232 ± 0.026 mV/ms; *p* = 0.292). During the post-TBS period, most of the recordings showed stable FV amplitudes before and after LTP induction, whereas the evoked responses were higher in the ANDRO group (control _fEPSP_: 0.277 ± 0.061 mV/ms, ANDRO _fEPSP_: 0.494 ± 0.068 mV/ms; *t*-test_(10)_ = 2.375, *p* = 0.038; Figure 6G). These data show that the more LTP generated in aged females with ANDRO is caused by more efficient neurotransmission but also by increased postsynaptic mechanisms during LTP induction in the ANDRO-treated group.

We also studied the attenuation phenomena in aged female degus (Figure 6H,I). At the beginning of the LTP induction, the ratio R2/R1 decreased by an increment in R1. This effect was similar between the control and ANDRO-treated group (control: 81.92 ± 8.26%, ANDRO: 63.99 ± 4.65%; *t*-test_(10)_ = 1.892, *p* = 0.088). The attenuation decay would depend on the pre- or postsynaptic mechanism involved. In aged females, the decay was comparable in both groups, where the R1 decreased to similar values (comparison last 10 min: Control: 90.09 ± 3.21%, ANDRO: 80.21 ± 3.05%; *t*-test_(10)_ = 2.231, *p* = 0.049). In both cases, the attenuation remained stable, because R1 remained higher until the end of the recording; however, the attenuation was higher in the ANDRO group, showing that LTP induces more stable changes in the postsynaptic terminals in aged females.

Altogether, the electrophysiological data show that ANDRO differentially affects basal neurotransmission in adult and aged female degus, but at both ages, it improves long-term potentiation.

### 2.4. Long-Term ANDRO Administration Differentially Modifies Pre- and Postsynaptic Hippocampal Proteins

**Figure 7:**
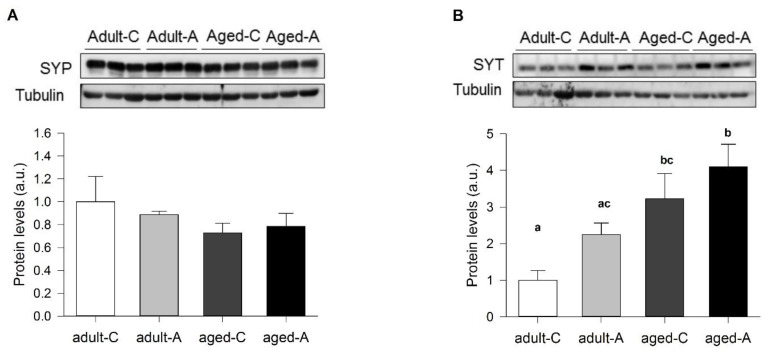
Differential effect of long-term ANDRO administration in the hippocampal presynaptic protein of adult and aged female degus. Densitometric analysis of presynaptic proteins: (**A**) SYP and (**B**) SYT of adult (36 months old) and aged (72 months old) control (C) and ANDRO (A) female degus. Data were analyzed statistically using one-way ANOVA, with the *p*-value indicated at the top of each figure. Different letters above bars show statistical differences between the same protein across the ANDRO treatments (Fisher’s LSD post hoc test). Results are expressed as mean ± SE (n = 3); a.u: arbitrary units.

Differences in synaptic activity observed in adult and aged females treated with the vehicle and ANDRO could partly be explained by the levels of different synaptic proteins. We performed Western blots to analyze the relative pre- and postsynaptic protein levels in the control and ANDRO-treated groups. Synaptophysin (SYP), a protein component of presynaptic vesicle membranes, showed no significant changes between the controls or when compared with the ANDRO treatment in both adult and aged degus (*p* = 0.516; Figure 7A). Instead, Synaptotagmin (SYT), a presynaptic vesicle membrane protein that acts as a Ca^2+^ sensor for fast exocytosis, showed significant results (F_(3,8)_ = 6.86, *p* = 0.013; Figure 7B). The comparison between the level of SYT in adults and the aged control was significant, being higher in aged than in adult females, suggesting the possibility of a compensatory mechanism for fast vesicle release in aged animals. Meanwhile, the treatment with ANDRO did not report differences within the groups of adults or aged females.

**Figure 8:**
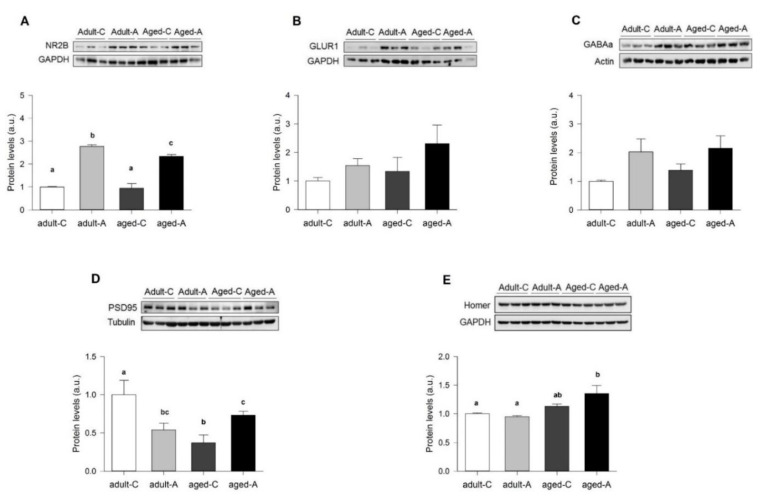
Differential effect of long-term ANDRO administration in the hippocampal postsynaptic protein of adult and aged female degus. Densitometric analysis of postsynaptic proteins: (**A**) NR2B, (**B**) GLUR1, (**C**) GABAA, (**D**) PSD95, and (**E**) Homer of adult (36 months old) and aged (72 months old) control (**C**) and ANDRO (**A**) female degus. Data were analyzed statistically using one-way ANOVA, with the *p*-value indicated at the top of each figure. Different letters above bars show statistical differences between the same protein across the ANDRO treatments (Fisher’s LSD post hoc test). Results are expressed as mean ± SE (n = 3); a.u: arbitrary units.

We also analyzed postsynaptic protein levels. We first measured the protein expression of the NR2B. Our result showed a significant effect through the different age groups (F_(3,8)_ = 62.07, *p* < 0.01), where ANDRO significantly increased the expression of NR2B in the hippocampus of adult and aged female degus (Figure 8A). Further, the α-amino-3-hydroxy-5-methyl-4-isoxazolepropionic acid (AMPA) receptor subunit 1 (GluR1) (Figure 8B) and the gamma-aminobutyric acid receptor (GABA_A_) protein expression (Figure 8C) did not vary with ANDRO treatments (*p* = 0.243 and *p* = 0.109, respectively). In the case of postsynaptic scaffold proteins, we analyzed the PSD95, which regulates the trafficking and localization of AMPARs and NMDARs, and we found a statistically significant effect of ANDRO treatment (F_(3,8)_ = 16.89, *p* < 0.01; Figure 8D). In particular, ANDRO reduced the protein expression of PSD95 in adult females while increasing the protein levels in aged females (Figure 8D). We also measured the expression of Homer1, showing a significant effect on the different age groups (F_(3,8)_ = 5.90, *p* = 0.020; Figure 7E). In particular, the increased expression of Homer1 and PSD95 in aged females suggested a robust postsynaptic structure due to ANDRO treatment. More studies should be performed to evaluate this hypothesis.

## 3. Discussion

This study aimed to evaluate and compare the effect of a long-term ANDRO treatment (12 months) on behavioral, electrophysiological, and molecular features obtained from adult and aged female degus. Our data demonstrated that ANDRO improves memory and preference for novel experiences in both ages. Neither age nor treatment affected the socialization of degus; however, ANDRO restored the defects in social recognition and long-term memory in aged female degus. On synaptic physiology, ANDRO showed different effects, either reducing the synaptic efficacy of basal neurotransmission in adult females or increasing it in aged females compared to their age-conspecific control. However, in both cases, ANDRO increases LTP, apparently through different mechanisms. Meanwhile, the analysis of synaptic proteins showed age-dependent changes in SYT, NR2B, and PSD95, which could explain some of the cognitive and physiological changes observed in females.

The use of *Octodon degus* as a neurological model has been extensively reported [5,10,11,12,14,15,16,17,18,19,20]. The broad range of degus’ behavioral complexities, the similarities with the human aging process, and the biological mechanisms underlying synaptic deficiencies make degus a multifaceted model. Furthermore, degus have demonstrated strong sex-dependent specificities in the aging process and the natural adaptation to respond to ambient variations [10,12,18].

Studies with females in scientific publications are commonly underrepresented. Most scientific research uses males, but some relevant differences have been missed in looking for similar features. In that context, we recently demonstrated that female degus, not males, suffer age-dependent synaptic detrimental effects [12,16]. Compared to males, young females (12–24 months old) showed more efficient synaptic transmission [10,12], but aged females (60–84 months old) displayed a significant reduction in synaptic efficacy compared to males. Moreover, aged females have fewer functional axonal terminals, needing the recruitment of twice as many axons to reach equivalent LTP levels as aged males [12]. These data demonstrate that age affects mainly females, despite no significant differences between male and female degus in brain asymmetry, size, and morphology of hippocampal areas having been found [20]. In addition, the cognitive evaluation of behavioral tasks showed that young females with high short-term memory performance exhibited a poor display of long-term memory tasks. In contrast, aged females with poor short-term memory displayed high performance in long-term memory tasks [16]. These data indicate that the mechanisms for short- or long-term memories are not preserved equally in females.

Here, we compared behavioral, physiological, and molecular aspects of adults and aged female degus. We studied whether some of the changes observed in aged females were evident in a previous adult stage, and how these could be modulated with long-term ANDRO administration. The reason for longer treatments is that shorter ones partially did not succeed in complete response. ANDRO improved synaptic strength in aged male degus and reduced several AD-related hallmarks, such as Aβ or tau protein [5,21,22], but did not increase LTP [5]. In that study, ANDRO was applied for only three months, perhaps not enough time to modify the mechanisms involved in LTP generation. In the AD-presymptomatic J20 mice model, four months of ANDRO was indeed enough to improve LTP in adult mice [9], the comparison between transgenic mice and the natural long-lived degus may not be appropriate, since the mechanisms involved could not be similar. In the present work, female degus were injected with ANDRO for over 12 months, and we observed that LTP increased over the control measurements in both adult and aged females. To our knowledge, this is the first long-term study of a long-lived animal. These data show that when working with a long-living animal model, the treatment duration and the age of the animals are the main factors to be considered when using ANDRO.

Multiple neurophysiological effects have been associated with the treatment of ANDRO, and controlling these during long-term use is far from easy. A summary of clinical studies using Andrographolide showed that more investigation is required considering dosage and secondary effects, including toxicity [23]. Some studies have reported side effects associated with high doses (4–6 mg/kg) causing allergic reactions, tiredness, headache, pruritus/rash, diarrhea, nausea, metallic taste, bitter taste, dry tongue, eyes sensitive to light, and decreased short-term memory [24,25]. However, the ANDRO dose used in the present study (2 mg/kg) was previously reported on other animal models [6], even in degus [5]. During its administration, we did take care to strictly follow the guidelines of animal supervision and care established by the bioethics committee. We observed no side effects throughout the study; thus, no animal was removed, indicating that our female degus tolerated ANDRO treatment with no evident toxic effects.

We used different behavioral paradigms to measure specific aspects of memory processing. As shown, tests such as NOR/NOL and the three-chamber social interaction test, which measure short-term recognition memory and social memory, respectively, demonstrated that ANDRO improved the exploration of new behaviors in adult and aged females, while enhancing the social recognition deficit in aged females. These aspects are considered relevant in the biological life of the animals: the recognition of changing environmental conditions and familiar conspecifics is essential for animal survival in natural situations. Studies from Uekita and Okanova suggested that the degu hippocampus, a key brain area being affected by aging, plays an important role not only in spatial recognition but also in social recognition [26]. The authors showed that changes in social behavior of degus resulting from hippocampal lesions, were interpreted as the impairment of social recognition rather than in novelty detection. Our results follow these statements, since social and object recognition memory, but not sociability, was impaired by age. Interestingly, ANDRO improved social and object recognition memory in aged female degus, suggesting that it modifies memory mechanisms. On the other hand, we performed the Barnes maze test, a highly hippocampal-dependent spatial learning task used to measure long-term memory [27]. During this test, we observed that the ANDRO-treated animals reduced the latency to the first visit and increased the time spent in the escape quadrant, showing that long-term memory processes were enhanced with the treatment. Altogether, these data indicate that ANDRO improves the performance of behavioral task-relevant aspects affected during the aging process.

Using brain slices of the adult and aged females, those treated long-term with ANDRO or the controls, we found interesting physiological aspects of synaptic communication. The facilitatory synaptic transmission in this synapse depends on the calcium availability that triggers synaptic vesicle Ca^2+^-dependent release. ANDRO did not affect this mechanism in adult or aged females during basal transmission. Noteworthy, the level of SYT, the presynaptic Ca^2+^ sensor, was higher in aged females and probably increased the fast vesicle release. We also measured the facilitation during LTP generation, the so-called attenuation. During LTP development, modifications in synaptic transmission involved postsynaptic and presynaptic components. The facilitation of the two pulses (R2/R1) mainly decreases, because R1 increases beyond the increment of R2; this effect is called “attenuation” of the R2/R1 ratio and can be permanent or transient along the development of LTP. Surprisingly, in adult females, the attenuation was short and similar to that previously observed in young female degus [12], suggesting that the attenuation mechanism in females is altered from very early in life. However, in adult females treated with ANDRO, the attenuation becomes stable, suggesting that the pre-and postsynaptic mechanisms involved in attenuation become ‘stabilized.’ This effect of ANDRO has been previously observed in mice [9] and suggests that ANDRO induces permanent changes in the hippocampal synaptic apparatus. More studies are required to demonstrate this hypothesis.

We also observed significant improvement in the LTP of these animals’ brain slices, indicating that the processes that involve acquisition, consolidation, and long-term retrieval improved with ANDRO. Nevertheless, the protocol to generate LTP appears to activate different mechanisms in adults and aged females. In adult females, the TBS induced the recruitment of more axons in the ANDRO-treated degus that evoked similar responses to the adult control and could be the reason for the higher LTP. Instead, in the aged females, the TBS recruited fewer axons that evoked higher EPSPs than the aged control. These data suggest that in aged animals, ANDRO treatment led to structural changes that made the synaptic transmission more efficient, influencing the postsynaptic mechanisms that induce plasticity.

Measurements of basal synaptic activity reported that adult females with ANDRO showed less excitable and efficient synaptic transmission than controls. A clue that can help to explain these differences may be relay in synaptic proteins. The treatment with ANDRO significantly reduced the expression of PSD95 in adults, whereas it increased NR2B-containing NMDARs. As a scaffolding protein, PSD95 stabilizes postsynaptic receptors contributing to spine morphogenesis and maturation [28,29], and it is high in young female degus compared to aged ones [5]. In the case of NR2B-type receptors, its increment correlates with the LTP increment [30]. NR2Bs receptors, generally allocated in extrasynaptic sites, are more highly mobile than the less mobile NR2As at synaptic sites [31]. Whether ANDRO regulates the lateral movement of these receptors is unknown, but as recently observed, the remotion of PSD95 from the synapses leads to an increase in the surface mobility of glutamate receptors [32]. Here, we did not examine the expression of NR2A-type receptors, but previously, we did show that its level diminished in aged compared to younger females [5]. The increment in the level of NR2B-type receptors suggests that this receptor could mobilize more to synaptic sites. Thus, lowering PSD95 expression could be a mechanism used by ANDRO to regulate the excessive NR2B expression that by itself can increase LTP [30,33]. This mechanism also could explain the lower basal activity in adult females. Instead, in aged females, ANDRO increased NR2B and PSD95, as well as NR2A, as was shown before [5], suggesting that these molecules are needed to stabilize an aged synapse. The high expression of Homer1 supports this idea in aged females with ANDRO, a component of postsynaptic density proposed not only as a scaffold protein but as a crosslinker for different synapse-related proteins [34].

The above-exposed antecedents show that ANDRO modulates several aspects of synaptic physiology. ANDRO is a non-ATP-competitive, substrate-competitive inhibitor of GSK-3β. The specific inhibition of GSK-3β by ANDRO induces the accumulation of β-catenin in the nucleus, causing the transcription of Wnt target genes [35], which is the central mechanism of canonical Wnt pathway activation. Interestingly, in the brain, ANDRO does not activate other signaling pathways that also increase the levels of Ser9-GSK-3β when active, such as Akt and mTOR, suggesting that its principal mechanism is mediated by the activation of Wnt signaling [35]. ANDRO also reduces astrogliosis, neuroinflammation caused by released cytokines, and oxidative stress in aged animals [8,21,36]. We did not assay for specific hallmarks of neurodegeneration or neuroinflammation in the present work. However, considering the age of the animals, we cannot dismiss the effect these aspects could have on the animal's physiological status, as was previously reported. It remains to be determined whether those aspects differ in timing and intensity among the sexes.

Our results suggested that as a long-lived animal, *Octodon degus* is an excellent model to visualize living social aspects, aging detrimental steps, and the sex component. On the one hand, young females appear to be more sensitive to social isolation stress lowering their synaptic efficacy, as compared to young males, despite their mechanisms of long-term memory appearing very stable, unlike males [10]. In normal conditions, young females show more efficient synaptic activity than males but similar levels of plasticity [12]. Compared to aged females, synapses in young females are highly efficient, but they generate similar LTP levels at both ages. Here, we showed that adult females are more similar to the younger ones in synaptic efficacy (compared to data in [12]) and better than aged females but with a similar level of LTP. Thus, the synaptic efficacy determined by how many axons are recruited to produce a particular postsynaptic evoked response can be modified, but it is different from what determines the amount of synaptic plasticity. On the other hand, the effect of ANDRO in females suggested that both mechanisms are modulated independently; in adults, the efficacy of synaptic transmission diminishes, but LTP increases several times. Instead, in aged females, both the synaptic efficacy and plasticity increase. Does the adult age represent an intermediate state for some aging features? A previous report showed a reduction in the content of SYP protein in aged compared to young females [5]. We did not find differences in adult and aged females, suggesting that lowering SYP is an earlier consequence of aging. In adult females, lower SYT and NR2B and higher PSD95 could induce a high-cost LTP, where two times as many axons are recruited with ANDRO but evoke the same postsynaptic response. In aged females, the interplay between high SYT and lower NR2B and PSD95 during basal activity may bring a place for plasticity: ANDRO can increase NR2B and PSD95, enhancing synaptic efficacy by reducing the recruited axons to evoke the maximum response. Therefore, long-term ANDRO administration could potentially improve females' cognitive impairment associated with normal aging.

## 4. Materials and Methods

### 4.1. Animals

Adult female degus (36 months old, n = 12) and aged female degus (72 months old, n = 12) weighing 200 ± 25 g and 209 ± 23 g (mean ± SEM), respectively, were obtained from our colony. These animals were all derived from laboratory-bred lines. Twenty-four female degus were used in this study: 12 animals in the ANDRO group (n = 6 adults, n = 6 aged) and 12 animals in the control group (n = 6 adults, n = 6 aged). Degus were kept in pairs of related and unrelated females housed in clear acrylic aquaria (length × height × depth: 50 × 35 × 23 cm) with bedding of hardwood chips, water, and food (commercial rabbit pellet; Champion, Santiago, Chile) provided ad libitum. Each cage contained 1 nest box made of clear acrylic (22 × 12 × 15 cm). Animals were kept in a ventilated room exposed to a 12:12 h light: dark cycle with temperatures controlled (yearly minimum = 13.4 ± 0.2 °C; yearly maximum = 24.9 ± 0.2 °C).

Intraperitoneal (IP) injections of 2.0 mg/kg of ANDRO (Sigma-Aldrich, Merck, Santiago, Chile) in the saline vehicle were administered 3 times per week, as described in the literature [5]. Control animals were injected with only vehicle solution. ANDRO and vehicle were given over 12 months. Each week, we measured body mass, and the doses for intraperitoneal injections were recalculated. To the best of our knowledge, this study is the first to evaluate the effects of ANDRO administered over a prolonged period in this long-lived rodent model.

Our design avoided the effect of hormonal fluctuation in 17–21-day regular cycling females by performing behavioral tests in the diestrus phase of the estrous cycle. All animal protocols followed the National Institutes of Health (NIH, Baltimore, MD, USA) guidelines and the National Institutes of Health guide for the care and use of laboratory animals (NIH Publications No. 8023, revised 1978). The Bioethical and Biosafety Committee approved all procedures of the Faculty of Biological Sciences of the Pontificia Universidad Católica de Chile (CBB-170113007-2017). The efforts were made to minimize animal suffering and reduce the number of animals used.

### 4.2. Behavioral Observations

As detailed below, adults and aged female degus, both control and ANDRO treated, were subjected to four behavioral tasks. The experiments were conducted from the least to the most intrusive, minimizing behavioral experiences' effect on the results. The experiments were as follows: (i) open field; (ii) the novel object recognition test; (iii) three-chambered social interaction test; (iv) Barnes maze test. Animals were subjected to one test daily (except the Barnes maze test, which is longer). All behavioral tests were performed during the active phase of the animals (between 09:00 to 16:00 h). At the end of each session, animals were returned to their home cages, and the area was cleaned with 70% ethanol solution.

#### 4.2.1. The Open-Field Test

To discard locomotor differences between groups and specifically evaluate the exploratory behavior, animals were observed for 5 min in the open-field test, consisting of a white Plexiglas box (length x height x depth: 100 × 100 × 100 cm). The frequency of total ‘central crossings’ (with a four-paw criterion) was scored [5]. In addition, the percentage of time in corners and in the middle arena, speed, and total length were assessed [5].

#### 4.2.2. Novel Object Recognition Test

The novel object recognition test is a double test used to evaluate cognition, particularly working memory and attention, and it can also test the preference for novelty in rodents [37]. The test arena used an open box (length × height × depth: 63 × 40 × 30 cm) made of white Plexiglas. For this test, we followed the object recognition protocol previously used in degus [5]. Briefly, animals were exposed to a 10 min familiarization assay and then tested in two consecutive five-minute assays, with a one-hour inter-trial interval. For Session 1 (Familiarization): two different objects (‘Object A’ and ‘Object B’) were placed in the corners of the home cage, and the animal was allowed to explore them for 10 min freely. Following this period, the test animal was returned to her home cage for one hour. Session 2 (‘Novel location recognition’ or NLR): one of the familiar objects (Object B) was moved to an adjacent unoccupied corner. The test animal was then free to interact with the objects A and B for five minutes. Following this period, the test animal was returned to her home cage for one hour. Session 3 (‘Novel Object Recognition’ or NOR): the familiar Object B was replaced by a new but similar object (i.e., similar texture and consistency). We recorded the time spent exploring each object (i.e., approaching within 1–3 cm of the object) and then calculated the recognition index (RI), which is the time spent with object B divided by the sum of the time spent with objects A and B, to quantify NLR and NOR.

#### 4.2.3. Three-Chambered Social Interaction Test

We used the three-chamber test to assess (i) social affiliation/motivation by comparing the time degus spent interacting with an empty cage versus a cage containing an unfamiliar (novel) degu; and (ii) social memory/preference for social novelty by measuring the time the degus spent interacting with an unfamiliar versus a familiar degu. The open-field arena was subdivided into three equal compartments using transparent Plexiglas walls, each with a small opening (2.8 cm in diameter) that allows access into each compartment. The degu to be used as the social partner was sex-matched, unfamiliar, and unrelated. The test comprised three sessions of 20-min. The social test was performed following the protocol previously described [38]. Briefly, three steps or phases were performed in the following order: Phase 1 or Habituation: the test animal was placed in the middle compartment and allowed to explore the apparatus. Phase 2 or Session 1 (‘Partner I’): the test animal was returned to the middle compartment; meanwhile, Partner I (a sex-matched, unfamiliar, and unrelated degu) was placed inside a wire cage in one of the lateral chambers. The test animal was free to interact with the social partner or an empty wire cage. At the end of Session 1, the test animal was returned to her home cage for one hour. Phase 3 or Session 2 (‘Partner II’): The test animal was returned to the middle compartment; a second unfamiliar, unrelated degu was placed inside an identical wire containment cage in the opposite side chamber, which was empty during Session 1. The test animal could freely explore between the first, already investigated, unfamiliar degu (Partner I) or the novel unfamiliar degu (Partner II). As measurements of social interaction, we recorded the time spent exploring the partner (i.e., the time the animal spent touching the containment cage housing or not housing the new partner for each chamber individually, with the forepaw or nose). The recognition index (RI) was calculated to evaluate social affiliation/motivation and social memory differences. The RI for Session 1 is the quotient of the time the degu spent with Partner I divided by the sum of the time spent with Partner I and the empty wire cage. For Session 2, the RI is the time spent with Partner II divided by the time spent with Partners I and II. A RI ≤ 0.50 indicates that degus lack social affiliation/motivation during Session 1 and social memory during Session 2.

#### 4.2.4. Barnes Maze Test

This test has a strong hippocampal-dependent spatial component [39], leading us to evaluate spatial navigation, learning, and memory [18]. The Barnes maze consisted of an elevated circular platform of white Plexiglas of 160 cm in diameter surrounded by a 45 cm-high wall. Eighteen circular holes (8 cm in diameter) were bored through the platform equidistant from each other (16 cm) and 5.5 cm from the outer edge. Every hole was blocked except for the target one. A plastic escape box (length × height × depth: 31 × 13 × 16 cm) was positioned under the escape hole. Accurate performance requires subjects to learn and remember the location of the escape hole; therefore, spatial cues (a combination of different colors and shapes: a yellow star, a red square, and a green apple) were placed on the wall of the maze [40]. Briefly, the procedure was divided into three phases: habituation, training, and a test phase, and it was performed as previously described [38,41]. Session 1 (Habituation): begins with the animal test into the escape box for two minutes. After that, the animal test was placed near the escape hole and left for one minute to escape. If the animal did not enter the escape box, it was gently picked up and put through the target hole into the escape box and left for two minutes. Finally, the animal was placed in the maze's center and left for four minutes to explore the platform and enter the escape box. If the animal did not enter the escape box, it was put into the escape box as mentioned above and left there for two minutes. Session 2 (Training): two days after Session 1, we trained each animal for 7 days. Session 3 (Test phase): seven days after Session 2, we exposed the test animals to a memory-retrieval session. Both the training and test phases consisted of four consecutive four-minute trials, separated by a five-minute resting phase in the animal home cage. At the beginning of each trial, the animal was confined for 30 s in a start box in the center of the maze. If the animal did not enter the escape box within the allotted time, it was manually picked up, placed in the escape box, and left undisturbed for 2 min. To discard locomotor differences between groups, we measured the speed and the distance (in meters) covered from the initiation of exploration of the escape hole to entrance into the escape hole. We registered the latency to the first visit of the escape hole, reference memory errors (every first visit to a non-escape hole in each trial), and working memory errors (repeated visits to the same non-escape hole in the same trial). 'Search strategies' used during retrieval trials were categorized into 3 groups: random, serial, and spatial, as previously described [10,42]. Briefly, searches were classified as ‘random’ when localized searches of escape holes were interrupted by central crosses, or when no systematic search pattern was discernible. ‘Serial searches’ were defined as searches of consecutive holes around the maze, and ‘spatial searches’ were defined as searches following a direct path to the escape hole. In all cases, a digital video camera (LifeCam Studio Full HD, Microsoft Corp, Redmond, WA, USA) was mounted above the test arena, and the performance of each animal was monitored with image tracking software (HVS Image, Hampton, UK).

### 4.3. Electrophysiology

Female degus were separated into two groups: adult (36 months old) and aged females (72 months old), control or treated with ANDRO. Every day, one animal was anesthetized and euthanized by decapitation. The brain was quickly removed and placed in a cold artificial cerebrospinal artificial (ACSF-)modified solution (sucrose replaces part of sodium), composed of the following (in mM): 85 NaCl, 75 sucrose, 3 KCl, 1.25 NaH_2_PO_4_, 25 NaHCO_3_, 10 dextrose, 3.5 MgSO_4_, 0.5 CaCl_2_, 3 sodium pyruvate, 0.5 sodium L-ascorbate, and 3 Myo-inositol (305 mOsm, pH 7.4, and oxygenated 95% O_2_/5% CO_2_). The brain was cut with a vibratome in coronal slices of about 300–350 µm containing the hippocampal formation. The slices were allowed to recover for an hour in the same solution at room temperature. Before recording, the solution was changed to an oxygenated ‘recording solution’ composed of (in mM): 126 NaCl, 3.5 KCl, 1.25 NaH_2_PO_4_, 25 NaHCO_3_, 10 dextrose, 1 MgSO_4_, 2 CaCl_2_, 3 sodium pyruvate, 0.5 sodium L-ascorbate, and 3 Myo-inositol (305 mOsm, pH 7.4, and oxygenated 95% O_2_/5% CO_2_). At the time of the recording, the temperature was raised to 35–36 °C. Each slice was placed under an upright infrared-differential interference contrast (IR-DIC) fluorescence microscope (Eclipse FNI, Nikon Instruments Inc., Melville, NY, USA) to visualize the hippocampal circuit with a 40x water objective. The Schaffer collaterals between CA3 and CA1 were stimulated using a bipolar concentric electrode (World Precision Instruments, Sarasota, FL, USA) connected to an ISO-Flex stimulus generator (AMPI, Jerusalem, Israel). The recording of evoked field excitatory postsynaptic potentials (fEPSPs) was performed with a glass electrode (World Precision Instruments, United States) of 0.5–1 MΩ pulled on a *p*-97 Micropipette Puller (Sutter Instruments, Novato, CA, USA) and filled with recording solution. The signals were recorded using a MultiClamp 700B amplifier (Axon CNS, Molecular Devices LLC, San Jose, CA, USA) and digitally sampled at 30 kHz using a Digidata-1440A interface (Axon CNS, Molecular Devices LLC, San Jose, CA, USA). The analyses were performed offline using pClamp 10.3 (Molecular Devices LLC, San Jose, CA, USA). Several protocols were used to characterize the basal status of this synapse: (i) The input–output curve, where increasing levels of current intensity were successively applied, and the fEPSP slopes and fiber volley (FV) amplitude measurements were obtained. The amount of current to evoke 60% of the maximum slope value was used to perform other protocols in the same slice. (ii) Paired-pulse facilitation (PPF) protocol, where two pulses, R1 and R2, were applied, separated by different inter-stimulus intervals (ISI), and the R2/R1 ratio was obtained to estimate the degree of facilitation. (iii) Long-term plasticity (LTP) protocol, where two pulses (R1 and R2, separated by 50 ms each) were evoked every 15 s. The first pulse (R1) slope was averaged for 15 to 20 min to obtain a stable basal signal (pre-TBS). Theta-burst stimulation (TBS, 5 bursts at 100 Hz every 20 s) was applied to induce LTP. The post-TBS was evaluated using the identical two pulses separated by 50 ms for at least 60 min. (iv) The attenuation was calculated using R1 and R2 pulses (%) and the PPF (R2-R1)/R1(%).

### 4.4. Western Blotting

The hippocampi of control and ANDRO-treated degus were dissected on ice and immediately processed as previously described [5,20,36]. Briefly, the hippocampal and cortical tissues were homogenized in RIPA buffer (10 mM Tris-Cl, pH 7.4, EDTA 5 mM, 1% NP-40, 1% sodium deoxycholate, and 1% SDS) supplemented with a protease inhibitor mixture and phosphatase inhibitors (25 mM NaF, 100 mM Na_3_VO_4_, and 30 μM Na_4_P_2_O_7_) using a Potter homogenizer and then sequentially passed through syringes of different calibers. The protein samples were centrifuged twice at 14,000 rpm for 20 min at 4 °C. The protein concentrations were determined using the BCA Protein Assay Kit (Pierce). The samples were resolved by SDS-PAGE, followed by immunoblotting on PVDF membranes. The membranes were incubated with the primary antibodies: glutamate ionotropic receptor AMPA type subunit 1 (GLUR-1, Santa Cruz Biotechnology, sc-13152, Santa Cruz, CA, USA), glycogen synthase kinase-3β (GSK-3β, Santa Cruz Biotechnology, sc-81462, Santa Cruz, CA, USA), GSK-3β phosphorylated in Serine 9 (p-GSK-3β, Santa Cruz Biotechnology, sc-373800, Santa Cruz, CA, USA), Homer (Santa Cruz Biotechnology, sc-17842, CA, USA), N-methyl D-aspartate receptor subtype 2B (NR2B, Santa Cruz Biotechnology, sc-365597, Santa Cruz, CA, USA), postsynaptic density 95 (PSD95, Thermo scientific, MA1-045), synaptotagmin I/II (SYT, Santa Cruz Biotechnology, sc-393392, Santa Cruz, CA, USA), synaptophysin (SYP, Santa Cruz Biotechnology, sc-17750, Santa Cruz, CA, USA) and gamma-aminobutyric acid receptor (GABA_A_ receptor, G0545, Sigma-Aldrich, Merck, Santiago, Chile). They were then washed with phosphate-buffered saline (PBS)-Tween 0.1% and incubated with secondary antibodies conjugated with HRP: anti-mouse, anti-rabbit, and anti-goat IgG peroxidase-conjugated antibodies (Pierce, Rockford, IL, USA). Later, the membranes were visualized using an ECL kit (Luminata Forte Western HRP substrate, Merck, Millipore, MA, USA). To analyze the results, all target protein signals were normalized against the loading control (α-tubulin, β-actin or GAPDH).

### 4.5. Statistical Analysis

All data are presented as the mean ± standard error (SEM). We used a *t*-test to analyze the effect of age in control female degus across each variable of the behavioral, electrophysiological, and/or biochemistry measurements. We used two-way ANOVAs to determine the effects of treatment (control and ANDRO), age (adult and aged degus), and the interaction between these factors. Fisher's LSD post hoc comparisons were performed to examine the individual main effect of treatment and age. The RI was analyzed in the NLR/NOR and the three-chambered social interaction tests. The assumptions of normally distributed data and homogeneous variances were confirmed using Shapiro–Wilk and Levene's tests, respectively. Statistical analyses were performed using the Statistica (StatSoft, Tulsa, OK, USA) software package. For the Western blot analysis, we used one-way ANOVA to analyze the effect of ANDRO treatment in both adult and aged degus. For the electrophysiological measurements, we used n = 6 adult females, n = 6 adult females + ANDRO, n = 6 aged females, and n = 6 aged females + ANDRO. At least three slices from each animal were considered replicates, averaged together, and counted like n = 1. Input–output curves were analyzed via two-way ANOVA to determine the effect of treatment and stimulus intensity, or age and stimulus intensity, and the interaction between the two factors. We plotted the FV amplitude value and its corresponding fEPSP slope to determine the differences in synaptic transmission efficacy. The points were adjusted with linear regression. To further compare whether the regression lines were different, we used the analysis of covariance (ANCOVA). This analysis tests the null hypothesis that both regressions are identical in slope and intercept. We applied this analysis to our data to determine the difference between our experimental groups. The PPF protocol was analyzed with two-way ANOVA to determine the effect of treatment and inter-stimulus interval, or age and inter-stimulus interval, and the interaction between the two factors. Using Pearson’s correlation, we found the correlation between FV amplitude and fEPSP slope before or after TBS. Using Fisher ’r’ to ’z’ transformation, we used z-scores to compute the significance of the difference when we compared two correlation coefficients. For the attenuation plot, we plotted the R1 (%) and R2 (%) and used the formula (R2-R1)/R1 (%). The two-way ANOVA determined the differences between time course and treatment, or time course and age, and the interaction between both factors. Additionally, an unpaired *t*-test was performed over the average of the last 10 min recordings to assay differences between the two groups. Amplitudes and area were obtained using PClamp 10.2, ( Molecular Devices LLC, San Jose, CA, USA) and the statistical analyses were obtained using the GraphPad Prism 5.1 (GraphPad software Inc., San Diego, CA, USA). Differences were considered statistically significant at *p* < 0.05.

## 5. Conclusions

Altogether, these data suggest that prolonged exposure to ANDRO improves complex behaviors, especially those related to age-related detrimental effects in females, enhancing exploratory behavior, social memory recognition, and synaptic mechanisms for basal and long-term memory processes.

## Figures and Tables

**Table 1:** Comparison of behavioral and molecular measurements between adult (36 months old) and aged (72 months old) control female degus.

	Age	
	Adult	Aged
***Open field***		
% Time in the central zone	14.73 ± 2.51	17.10 ± 5.78
% Time in corners	45.30 ± 3.85	42.72 ± 7.41
Number of central crossings	5.50 ± 1.08	5.33 ± 1.43
Total distance moved (m)	44.31 ± 6.38	35.44 ± 4.82
Average speed (m/s)	0.15 ± 0.02	0.12 ± 0.02
***NLR/NOR Test***		
RI NLR	0.61 ± 0.03	0.54 ± 0.05
Time with the moved object (s)	18.96 ± 3.88	21.29 ± 3.82
Time with familiar object (s)	28.81 ± 4.82	13.28 ± 3.34
RI NOR	0.62 ± 0.04	0.49 ± 0.04
Time with the new object (s)	14.77 ± 3.08	16.78 ± 6.25
Time with familiar object (s)	8.95 ± 2.11	15.88 ± 4.72
***Three-chambered social interaction test***		
RI session I	0.69 ± 0.06	0.700 ± 0.05
Time spent with partner I (s)	292.71 ± 32.95	148.39 ± 28.01
Time spent with empty wire cage (s)	117.08 ± 34.53	56.57 ± 7.90
RI session II	0.61 ± 0.04	0.45 ± 0.06 *
Time spent with partner II (s)	146.15 ± 16.41	94.57 ± 21.84
Time spent with partner I (s)	96.61 ± 17.55	103.33 ± 16.47
***Barnes maze***		
Time to find the escape hole	83.71± 29.13	182.35 ± 23.11 *
Percentage of time spent in the quadrant	57.72 ± 5.75	30.28 ± 5.50 **
Speed of travelling	0.20 ± 0.03	0.08 ± 0.01 **
Total distance travelled	13.22 ± 3.89	12.51 ± 1.38
Reference memory errors	9.04 ± 1.00	9.04 ± 1.18
Working memory errors	4.00 ± 1.39	2.25 ± 0.33
***Presynaptic proteins*** ^†^		
SYP	1.00 ± 0.22	0.73 ± 0.09
SYT	1.00 ± 0.26	3.22 ± 0.69 *
***Postsynaptic proteins*** ^†^		
NR2B	1.00 ± 0.02	0.94 ± 0.20
GluR1	1.00 ± 0.12	1.34 ± 0.48
GABAA	1.00 ± 0.03	1.39 ± 0.22
PSD95	1.00 ± 0.19	0.37 ± 0.10 *
Homer1	1.00 ± 1.27	1.13 ± 0.04 *

† The densitometric analysis is shown as relative values to the control. Abbreviations: SYP, synaptophysin; SYT, synaptotagmin; NR2B, N-methyl D-aspartate receptor subtype 2B; GluR1, glutamate receptor 1; GABA_A_, gamma-aminobutyric acid receptor; PSD95, postsynaptic density protein-95. Values are mean ± SEM. * *p* ≤ 0.05 and ** *p* < 0.01.

## Data Availability

The data that support the findings of this study are available in the methods and/or Supplementary Material of this article.

## Supplementary Materials

The following supporting information can be downloaded at: https://www.mdpi.com/article/10.3390/ijms24021105/s1, Figure S1: Effect of the long-term ANDRO administration on cognitive performance measured by the open-field test in adult and aged female degus; Figure S2: Effect of the long-term ANDRO administration on cognitive performance measured by the Barnes maze test in adult and aged female degus; Figure S3: Effect of the long-term ANDRO administration on the navigation search strategy utilized during training sessions on the Barnes maze test.
