# Supplementary material for: Age-Dependent Behavioral and Synaptic Dysfunction Impairment Are Improved with Long-Term Andrographolide Administration in Long-Lived Female Degus (Octodon degus)
Source: Int J Mol Sci. 2023 Jan 6;24(2):1105. doi: 10.3390/ijms24021105 (PMC9866633; doi:10.3390/ijms24021105)
Supplement: Supplementary file 1 [file ijms-24-01105-s001.zip › ijms-2041605-supplementary.pdf]

SUPPLEMENTARY MATERIAL

Latency to find the escape hole, number of reference and memory errors and the navigation strategy during the training phase on the Barnes maze test

Repeated measures analysis during the training phase, showed that the latency to find the escape hole in female degus treated with ANDRO or vehicle showed a significant effect of treatment [$F_{(1,154)} = 4.684$, $p = 0.032$], no effect of time ($p = 0.613$), but a significant interaction was found between the two factors [$F_{(6,154)} = 2.591$, $p = 0.020$ Fig. S2A]. On the other hand, when we compared adult and aged female degus, the latency to find the escape hole showed a non-significant effect of treatment ($p = 0.229$) or time ($p = 0.628$), but was altered by the interaction between the two factor [$F_{(6,154)} = 2.376$, $p = 0.032$; Fig. S2B].

We also measured the numbers of reference memory and working memory errors to find the escape hole during the training phase. For the reference memory errors, the repeated measures analysis in female degus treated with ANDRO or vehicle resulted in a non- significant effect of treatment ($p = 0.239$), a significant effect of time [$F_{(6,154)} = 3.531$, $p < 0.01$], and remained no significantly different across the interaction between both factors ($p = 0.638$; Fig. S2C). In general, we observed a significant decrease in the number of reference memory errors across day 6 and 7 of training. Similarly, in adult and aged female degus there was a non-significant effect of treatment ($p = 0.089$), a significant effect of time [$F_{(6,154)} = 3.521$, $p < 0.01$], and there was not significant interaction between both factors ($p = 0.889$; Fig. S2D). Similar both adult and aged degus made significantly less reference memory errors across the last days of training.

On the other hand, for the number of working memory error in female degus treated with ANDRO or vehicle, we found no effect of treatment ($p = 0.553$), time ($p = 0.088$), nor was by the interaction between the two factors ($p = 0.924$; Fig. S2E). Whereas, when we compared adult and aged female degus the repeated measures analysis showed a non-significant effect of treatment ($p = 0.119$], time ($p = 0.086$), and remained no significantly different across the interaction between both factors ($p = 0.994$; Fig. S2F).

Lastly, throughout the training period, the strategy used by aged female degus treated with vehicle during day 1 consisted of mostly serial-random strategy (46% and 42% respectively; Fig. S3B). By the end of training sessions, we observed that aged female degus adopted mostly a random-oriented pattern (88%; Fig. S3B). On the other hand, aged females treated with ANDRO were characterized by largely serial and random-oriented strategy (50% and 42% respectively; Fig. S3D). At the end of the training session, aged female degus acquired a combination of mostly serial (62%) strategy with almost similar proportion of random (21%) and spatial strategy (17%; Fig. S3D). Instead, adult females treated with vehicle applied mostly random and serial-oriented strategy (58% and 29% respectively; Fig. S3A) during day 1, changing to serial and random-oriented strategy by the end of the training period (58% and 25% respectively; Fig. S3A). In contrast, adult female treated with ANDRO, during day 1 of training, altered their search strategy from a combination of random (79%) and serial (21%) strategy (Fig. S3C) to a mostly serial (50%) with spatial and random strategy used in the same proportions (25 and 25% respectively; Fig. S3C) by the end of training sessions.

Figure legends

Figure S1. Effect of the long-term ANDRO administration on cognitive performance measured by the Open field test in adult and aged female degus. (A) time spent in the corners (B) time spent in the central zone of arena (C) number of central crossing (D) speed and (E) total distance travelled. The data were analysed statistically using two-way ANOVA followed by Fisher's LSD post hoc test. The effect of treatment, age and the interaction between the two factors are indicated in the top of the figure. Each symbol corresponds to data from a single-age treatment group, represented as the mean ± SEM obtained from n = 6 adult females, n = 6 adult females + ANDRO, n = 6 aged females, and n = 6 aged females + ANDRO.

Figure S2. Effect of the long-term ANDRO administration on cognitive performance measured by the Barnes maze test in adult and aged female degus. (A-B) learning curve of latency of the first visit to escape hole (C-D) learning curve of the reference memory errors (E-F) learning curve of working memory errors. Results are expressed as mean ± SEM. n = 6 adult females, n = 6 adult females + ANDRO, n = 6 aged females, and n = 6 aged females + ANDRO.

Figure S3. Effect of the long-term ANDRO administration on the navigation search strategy utilized during training sessions on the Barnes maze test. S: serial strategy; E: spatial strategy; R: random strategy. (A) adult female degus (B) aged female degus (C) adult female degus + ANDRO (D) aged female degus + ANDRO. The 'random strategy' was defined as searches with no systematic search pattern or when searches of scape hole were interrupted by central crosses. The 'serial searches' were defined as searches of consecutive holes around the maze, and 'spatial searches' were defined as searches following a direct path to the escape hole.

Figure S1

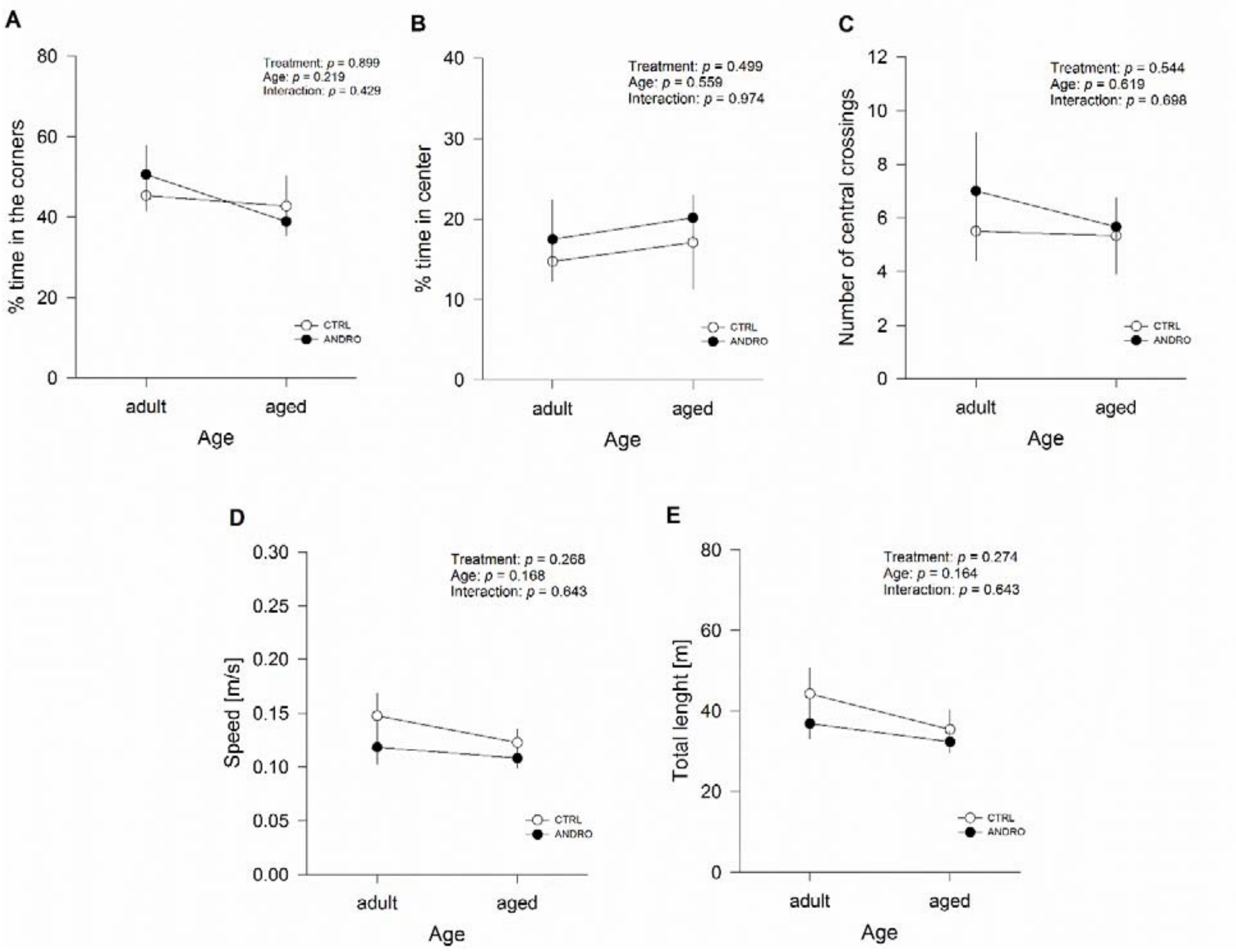
A
% time in the corners
Treatment: p = 0.899
Age: p = 0.219
Interaction: p = 0.429
CTRL
ANDRO
adult
aged
Age
B
% time in center
Treatment: p = 0.499
Age: p = 0.559
Interaction: p = 0.974
C
Number of central crossings
Treatment: p = 0.544
Age: p = 0.619
Interaction: p = 0.698
D
Speed [m/s]
Treatment: p = 0.268
Age: p = 0.168
Interaction: p = 0.643
E
Total lenght [m]
Treatment: p = 0.274
Age: p = 0.164
Interaction: p = 0.643

Figure S2

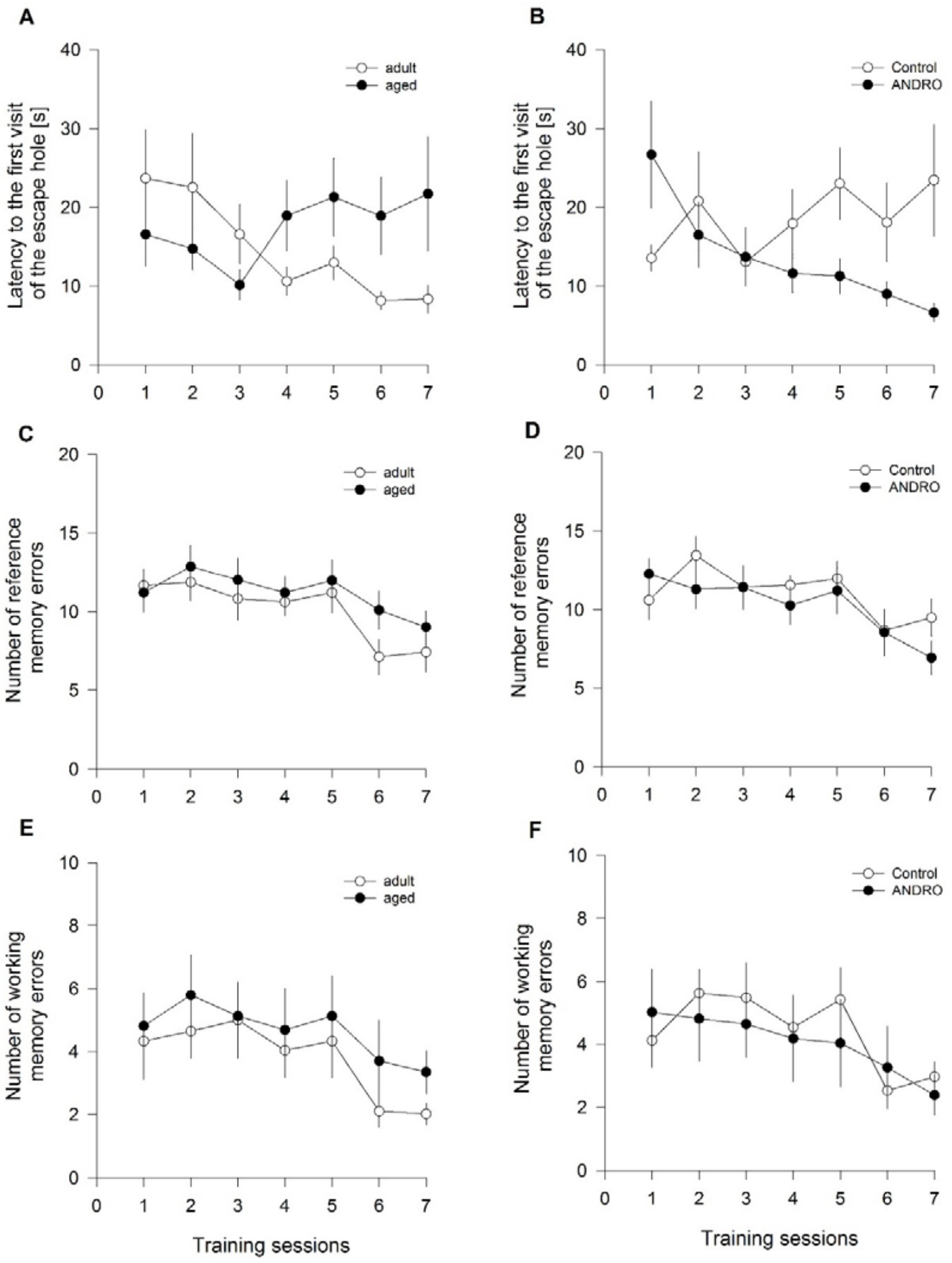
A
B
C
D
E
F
adult
aged
Control
ANDRO
Latency to the first visit of the escape hole [s]
Number of reference memory errors
Number of working memory errors
Training sessions

Figure S3

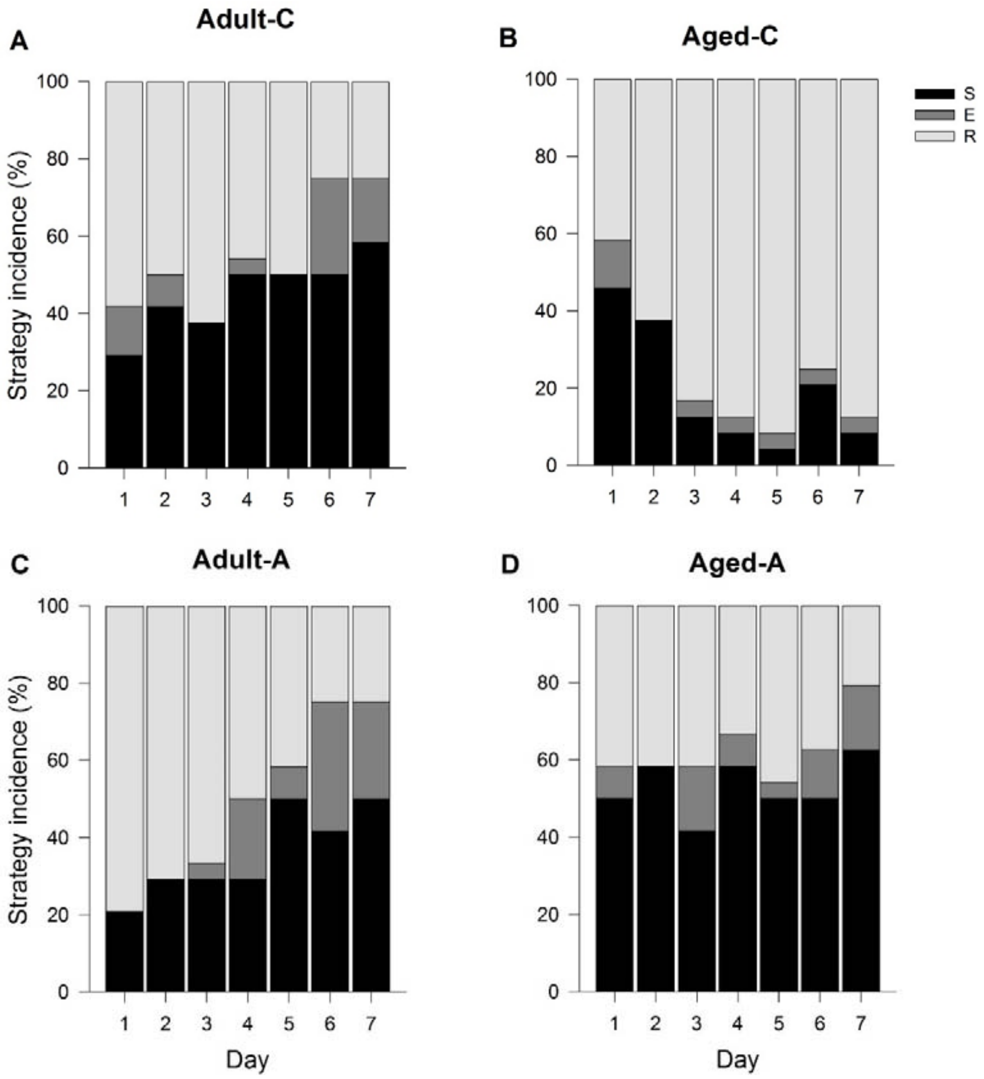
A
Adult-C
B
Aged-C
C
Adult-A
D
Aged-A
S
E
R
Strategy incidence (%)
Strategy incidence (%)
Day
Day
0
20
40
60
80
100
1
2
3
4
5
6
7
